# Prediction of crime occurrence from multi-modal data using deep learning

**DOI:** 10.1371/journal.pone.0176244

**Published:** 2017-04-24

**Authors:** Hyeon-Woo Kang, Hang-Bong Kang

**Affiliations:** Dept. of Digital Media, Catholic University of Korea, Bucheon, Gyonggi-Do, Korea; University of Texas at San Antonio, UNITED STATES

## Abstract

In recent years, various studies have been conducted on the prediction of crime occurrences. This predictive capability is intended to assist in crime prevention by facilitating effective implementation of police patrols. Previous studies have used data from multiple domains such as demographics, economics, and education. Their prediction models treat data from different domains equally. These methods have problems in crime occurrence prediction, such as difficulty in discovering highly nonlinear relationships, redundancies, and dependencies between multiple datasets. In order to enhance crime prediction models, we consider environmental context information, such as broken windows theory and crime prevention through environmental design. In this paper, we propose a feature-level data fusion method with environmental context based on a deep neural network (DNN). Our dataset consists of data collected from various online databases of crime statistics, demographic and meteorological data, and images in Chicago, Illinois. Prior to generating training data, we select crime-related data by conducting statistical analyses. Finally, we train our DNN, which consists of the following four kinds of layers: spatial, temporal, environmental context, and joint feature representation layers. Coupled with crucial data extracted from various domains, our fusion DNN is a product of an efficient decision-making process that statistically analyzes data redundancy. Experimental performance results show that our DNN model is more accurate in predicting crime occurrence than other prediction models.

## Introduction

The prediction of crime occurrences [1–7] has received considerable attention on account of its prospective benefits. This predictive capability would notably contribute to effective police patrols. According to the 2014 Chicago crime record, there were a total of 274,064 incidents of crime in 2014 and an average of 750 cases per day in that city. The results of these crimes, including injuries and deaths, are very serious. Fundamental crime prevention requires the strengthening of patrols, which is costly in terms of financial and human resources. Furthermore, the above records show that patrols are simply undertaken depending on the location of known crime-ridden districts or the empirical knowledge of police. One possible approach to solve this problem is accurately predicting the probability of crime occurrences at a given date and location by scrutinizing and modeling various previous data on criminal activities. The prediction results would enable police to perform an effective predictive police patrol in so-called crime hotspot areas.

The occurrence of crimes has been the subject of many studies. Several studies have focused on identifying patterns among criminal incidents. These studies found spatial and temporal crime occurrence patterns. They additionally demonstrated the relationship between the occurrence of a crime and information about the surrounding area. Furthermore, a few recent studies have employed crime-occurrence report data as well as additional crime occurrence information from multiple domains such as demographics, housing, economics, education, and weather [1, 8–17]. These studies produced successful results in predicting crime.

Conventional methods have used structured data on surrounding areas, such as population, race, income, and education from multiple datasets. However, they have not considered environmental context information. The motivations for using environmental context information are Broken Windows Theory (BWT) [18] and Crime Prevention Through Environmental Design (CPTED) [19, 20]. These studies demonstrated that neighborhood appearance (which we call environmental context in this paper) affects criminal activity, i.e., it is implied that environmental context information is directly related to crime occurrence. To provide environmental context information for our prediction model, we used image data collected from Google Street View. Because image data has an unstructured data form, conventional methods, which can use only structured data, cannot deal with image data. Furthermore, these methods handle multiple datasets equally. These methods result in limitations in predicting crime occurrences because of nonlinear relationships, redundancies, and dependencies between multiple datasets. In other words, to accurately predict crime occurrences and ultimately enhance the accuracy of crime prediction models, it is necessary to effectively fuse multi-modal data according to deep learning and to consider environmental context information. To solve this problem, we employ a deep neural network (DNN) with feature-level data fusion. The environmental context feature group is a feature vector extracted by convolutional neural network (CNN) using image data. We divide structured data into spatial and temporal feature groups, and each feature group is fed independently into DNN for feature learning. Three feature-learning results are fused into joint feature representation layers.

**Fig 1:**
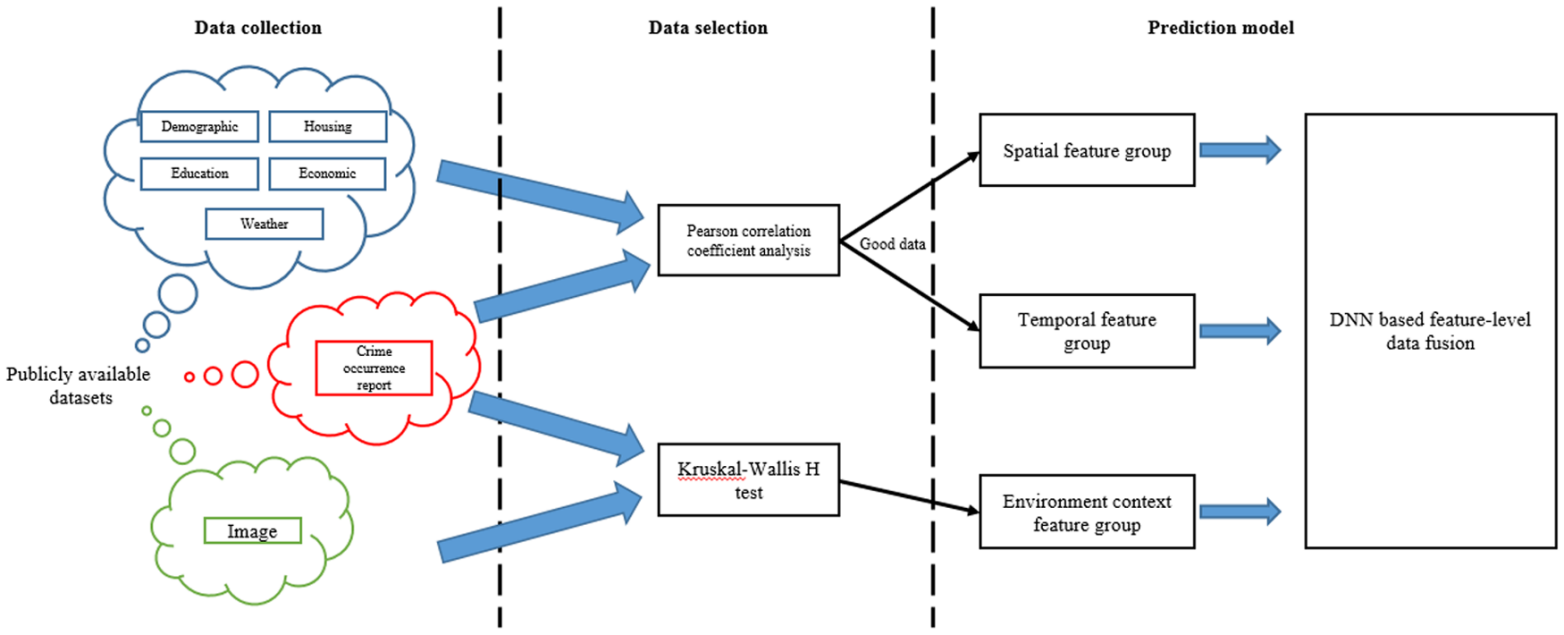
Overview of the proposed approach. Our approach consists of three steps. First data collection step, second data selection step, and third prediction model learning step.

In this paper, we propose a crime occurrence prediction method that considers environmental context information using multi-modal data fusion. Fig 1 depicts an overview of our approach.

Our approach consists of three structural components based on data generated from the prediction of crime occurrences. (1) Data collection: for prediction of crime occurrence, we collect the data from various online databases. We collected crime occurrence reports from the City of Chicago Data Portal; demographic, housing, education, and economic information from American FactFinder; and weather data from the Weather Underground. Furthermore, we captured image data for environmental context information from Google Street View. (2) Data selection: data selection is conducted to select crime-related data for crime prediction by analyzing the relationship between crime occurrences and the collected data. To select crime-related data, we analyzed the relationships between crime occurrences and collected data by conducting statistical analyses. This process facilitated accurate and effective prediction of crime occurrences, as outliers degrade prediction performance. (3) Prediction model: as our prediction model, we propose a multi-modal fusion approach to enable accurate crime occurrence prediction. A DNN-based prediction model with feature-level data fusion generally performs more accurately than the direct concatenation method [21]. We therefore used the DNN-based prediction model with a feature-level data fusion method. The DNN we employed in our method consists of the following four layer groups: spatial, temporal, environmental context, and joint feature representation layers. The main contributions of our paper are summarized below.

- We describe our approach to collecting various types of data from publicly available datasets. In addition, we introduce the concept of environmental context information.
- A method based on a statistical test is proposed to enable selection of crime-related data. Additionally, we demonstrate the relationship between environmental context information and crime occurrences using a statistical approach.
- We obtain accurate prediction performance by developing a deep-learning-based multi-modal data fusion approach. Our prediction model shows state-of-the-art performance.

## Related work

In this section, we explore several factors related to crime, crime prediction methods, deep learning, and environmental context information.

### Factors in crime occurrence

#### Urban factor

Early studies in criminology have tried to demonstrate the relationship between crime and various influencing factors such as demographics [1], economics [10, 14, 22], and unemployment [12, 13, 15]. Even recently, these studies are being continued to demonstrate relationships between crime and various influencing factors. For example, Kelly [14] considered the relationship between inequality and crime for urban counties in the USA and demonstrated that socially disadvantaged people committed most violent crimes; the study concluded that the most disadvantaged members of society who lived in areas of high inequality faced greater pressure and incentives to commit crime, leading to committing violent crimes. Hojman [12] studied inequality, unemployment, and crime in Latin American cities by taking account of the diversity among cities and using a regression analysis that indicated the role of deterrents, poverty, and inequality as causes of crime. Poveda [10] studied socio-economic and violent crime in seven cities in Columbia. Their analysis showed that cities’ economic deprivation and high population density are strong factors in homicide rates. Additionally, they found that economic growth, inequality, poverty, and human capital had a negative influence on violent crime. Alves et al. [1] studied the relationship between homicide and 11 urban factors with data from Brazilian cities. Their study found that GDP, GDP per capita, income, and male population have a positive correlation with homicide, while child labor, elderly population, female population, illiteracy, sanitation, and unemployment have a negative correlation with homicide.

#### Spatial and temporal patterns

Various studies have explored patterns of crime occurrence. These patterns can be divided into two categories: spatial and temporal patterns. Spatial patterns denote the location of the occurrence of crime; for example, downtown, residential districts, and entertainment districts, and temporal patterns are time periods and seasonality of the occurrence of crime.

Spatial pattern analysis of crime is one of the classical approaches. Cusimano et al. [11] analyzed spatio-temporal patterns of violent injuries in Toronto, Canada using a multivariate Poisson regression model. To analyze the location of injury occurrences, they used the emergency medical services (ambulance dispatch) dataset of Toronto. They found hotspots for ambulance dispatches such as entertainment districts in the time period from 0:00 to 3:59, which includes the closing time of bars. Their results showed that most injuries and crimes occurred around bars, clubs, and a few residences. Mohler et al. [6] identified crime patterns in which crime occurrences spread from an initial crime location to surrounding areas, similar to patterns created by earthquake aftershocks. They utilized an earthquake aftershock prediction algorithm (epidemic-type aftershock sequences) and self-exciting point process modeling using historical crime records for crime prediction. Other studies have investigated the connection of criminology and epidemiology. Akers and lanier [23] explained disciplinary commonalities and differences between criminology and epidemiology. As a result, they proposed a new paradigm called epidemiological criminology which links epidemiological approaches with criminology.

There are two theories about the relationship between seasonality and crime [24, 25]. (1) Temperature/aggression theory: the occurrence of crime increases in the summer because of high temperatures [26]. This theory claims that people’s behavior becomes aggressive owing to the high temperatures of the summer, leading to the increase of crime occurrences. (2) Routine activity theory: this theory asserts that people’s behavior becomes aggressive owing to the high temperatures but does not cause an increase in the occurrence of crime [9]. Routine activity theory holds that people are more active in warm weather, which leads to increased human interaction, ultimately suggesting that the opportunity for crime occurrence rises. However, some studies have not found patterns of seasonality in crime occurrence [17, 27].

### Crime prediction method

Crime prediction methods have utilized a variety of machine learning techniques, such as regression analysis [2], kernel density estimation (KDE) [3], and support vector machine (SVM) [28], and data, such as statistical data [2, 7, 29] and social media data [3, 30].

Liao et al. [5] built a Bayesian-based crime prediction model using geographical information and victim characteristics. They divided characteristics of crime sites into two regional types: private regions and public regions. Next, they used a discrete distance decay function to create a geographic profile, which is the probability distribution of crime occurrences. Finally, to accurately predict the location of the next crime occurrence, geographic profiles were combined with Bayesian learning theory. Gorr et al. [4] proposed a short-term crime prediction method using a one-month time horizon. They used five types of crime statistical data (aggravated assault, burglary, drugs, robbery, and simple assault) obtained from 1990 through 1998 in Pittsburgh, Pennsylvania. Additionally, they used demographic, social, and education information from the 1990 Pittsburgh census. In their study, they compared ten statistical analysis methods, including regression and time-series analysis. They found seasonal patterns in crime occurrences; i.e., general crimes are more concentrated in the summer on account of increased social interaction, while burglaries and robberies are more prevalent in cold weather on account of seasonal economic pressures, including unemployment. Similarly, Chen et al. [2] applied an autoregressive integrated moving average model for short-term crime prediction in a city in China. The model used is a well-known time-series analysis method for predicting future events. Shingleton et al. [29] used an approach based on regression analysis to predict three crime types (violence, homicide, and assault) in Salinas, California using ordinary least squares, Poisson regression, and negative binomial regression models. Their experimental results confirm similar performance among these three models. However, Poisson regression and negative binomial regression models require the assumption that data follow the Poisson distribution. If the data cannot be fitted using a Poisson distribution, ordinary least squares is sufficient.

For prediction of crime hotspots, Kianmehr and Alhajj [28] proposed a computational framework for application in Columbus, Ohio and St. Louis, Missouri using SVM with k-means clustering. They highlighted the lack of negative samples in many types of datasets and addressed this problem using k-means clustering to partition the dataset into small sets. In their framework, these small sets are labeled as either a hotspot class or non-hotspot class according to the respective crime rate. A dataset with a crime rate higher than a predefined crime rate is labeled as a hotspot class (positive); otherwise, it is labeled as a non-hotspot class (negative). Their framework is universally applicable to crime hotspot prediction and other prediction task domains (i.e., general frameworks). Furthermore, Wang et al. [7] used SVM for the prediction of criminal recidivism. They used datasets from the National Archive of Criminal Justice Data of the Inter-University Consortium for Political and Social Research. They evaluated the performance of their SVM by training a logistic regression and multi-layer neural network. Although the SVM and multi-layer neutral network showed similar performance, they both outperformed the logistic regression method. As a result, the authors combined predictions from the three models and obtained the best performance.

Recently, some studies have used social media data and KDE for crime prediction [3, 30]. Gerber [3], for example, predicted crimes using social media information (e.g., GPS-tagged tweets). He hypothesized a pattern that determines that crime-related tweets dramatically increase in areas surrounding the locations and times of crime incidents. He analyzed the topics of tweets by using a latent Dirichlet allocation topic model. He then trained the prediction model and obtained successful results. However, this method required a collection of tweets. Although a collection of tweets is free, obtaining a collection of historical tweets is either impossible or would require financial expenditures.

Despite the above contributions, most existing methods treat data from multiple domains on an equal footing, such as by directly concatenating features or performing a weighted summation. These approaches do not consider the different characteristics of data from multiple domains, which can be problematic. Thus, we use an alternative method to appropriately fuse data from multiple domains.

### Feature-level data fusion using deep learning

Deep learning has delivered notable performance in computer vision, such as image classification [31]. In addition, deep learning has been utilized for new feature representation and abstraction [32]. Using this mechanism, it can be easily applied to summarize key information or features from large amounts of data or complex data. Because deep learning is employed to learn from extensive data, the approach outperforms classical methods.

In computer vision, feature-level data fusion was conducted using deep learning in some studies. Lu et al. [33] fused global and detail images for quality assessment of image aesthetics, for which they built two CNN models. The first model was used for fully resized images; the other was used for fine-grained random cropping of images. Each CNN model extracted global and local features for image aesthetics. The extracted features were fused in the first fully connected layer. In addition, Liu et al. [34] proposed the fusion of RGB and depth images for 3D object detection. They represented joint features by feeding the extracted features into a bimodal deep Boltzmann machine, which was configured as a Gaussian-Bernoulli restricted Boltzmann machine. Finally, an exemplary SVM was trained using joint representation features. Moreover, Ngiam et al. [35] proposed a middle-level feature representation method between audio and video using deep auto-encoders. Their proposed model consists of joint representation and a multi-tasking structure.

### Environmental context information

We used environmental context information that was inspired by BWT [18] and CPTED [19, 20]. These two theories demonstrate a connection between neighborhood appearances and criminal activity. BWT argues that the disorganization of social environments, such as broken windows, litter, crashed cars, etc., is the cause of increased criminal activity. That is, a place of visually perceived disorder potentially has a high probability of occurrence of crime. CPTED is an urban planning and architectural design initiative that is intended to prevent crime and reduce fear of crime using the built environment. CPTED is designed along three principles, namely natural surveillance, natural access control, and territorial reinforcement. Many studies have been conducted to investigate the ability of CPTED to prevent crimes from occurring, successfully determining the factors responsible for the prevention of crime occurrences. After all, BWT and CPTED suggest that environmental context information has a relationship with crime. In practice, Salesses et al. [36] collected street-level images and measured the visually perceived safety, uniqueness, and wealth scores of images depicting urban places through pairwise comparison experiments with human visual perception (the Place Pulse 1.0 dataset). They found that the visually perceived safety score has a negative correlation with crime (i.e., safer appearance leads to less crime).

## Dataset

### Environmental context information

For example, consider locations *l*_1_ and *l*_2_ belonging to census tract *C*_*n*_, {*l*_1_, *l*_2_, ⋯, *l*_*m*_} ∈ {*C*_*n*_} If *l*_1_ is an ordered and clean location, such as modern buildings and clean parks, whereas *l*_2_ is a disordered and dirty location, such as industrial buildings and graffiti-marked alleys, then the probability of a crime occurring at the two locations is likely to be quite different. That is, certain locations have different crime occurrence probabilities according to environmental context information. However, prior studies used only information about the surrounding area, such as population, race, income, and education data using multiple datasets; they did not consider environmental context information. Therefore, to accurately predict crime occurrences, we should consider environmental context information.

**Fig 2:**
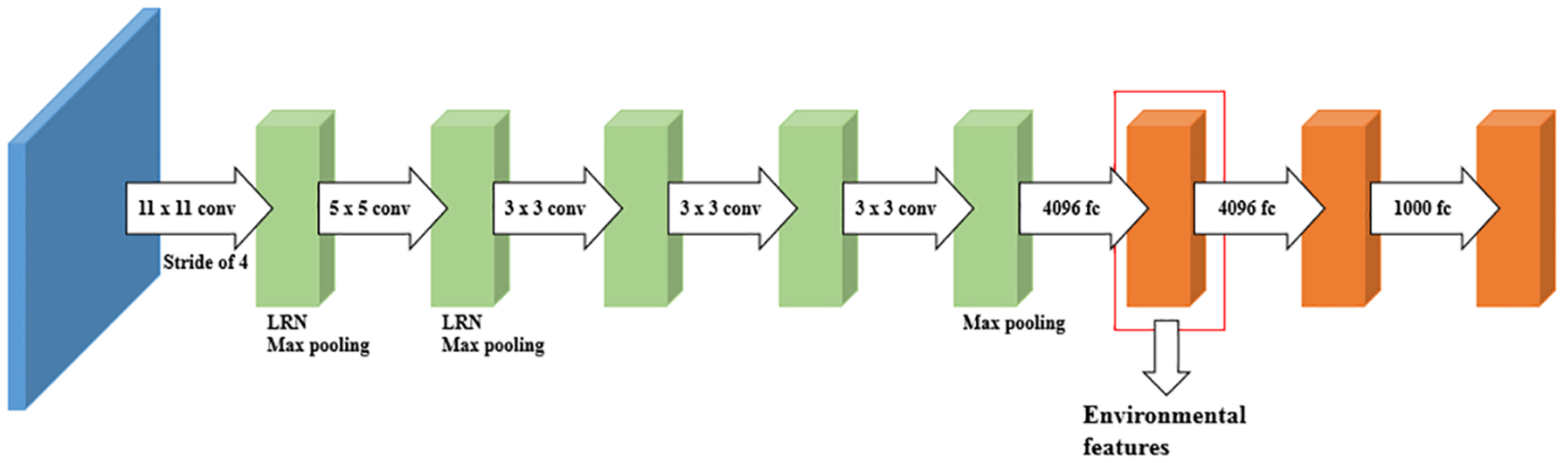
The structure of Alexnet. It consists of five convolutional layers and three fully connected layers. We used the results of the first fully connected layer as a feature vector.

Because image data is unstructured data, we extracted features from image data collected from Google Street View images to provide our prediction model with environmental context information (Image data are in dataset menu of the site http://cvml.catholic.ac.kr). Although the image data were not collected in real time, they are considered sufficient to provide the prediction model with relevant environmental context information. We extracted features from the image data by employing Alexnet [31], which showed good performance in terms of image classification. As shown in Fig 2, it consists of five convolutional layers and three fully connected layers. The first convolutional layer has 96 kernels of size 11 x 11 x 3 with a stride of 4. The first convolutional layer applies max pooling and local response normalization. The second convolutional layer has 256 kernels of size 5 x 5 x 96, and it also applies max pooling and local response normalization. The third and fourth convolutional layers have 384 kernels of size 3 x 3 x 256 and 3 x 3 x 384, respectively. The results of the third and fourth convolutional layers have no max pooling and local response normalization applied. The fifth convolutional layer has 256 kernels of size 3 x 3 x 384. The fifth convolutional layer only applies max pooling. The three fully connected layers have 4096, 4096 and 100 neurons, respectively. The image data were provided to Alexnet trained on ILSVRC 2012 (http://www.image-net.org/challenges/LSVRC/2012/) data, and we used the results of the first fully connected layer as a feature vector (4096-D). Consequently, the results of the first fully connected layer of Alexnet were used as environmental context features in our prediction model (i.e., pre-trained Alexnet was used as a feature extractor).

### Data collection

**Fig 3:**
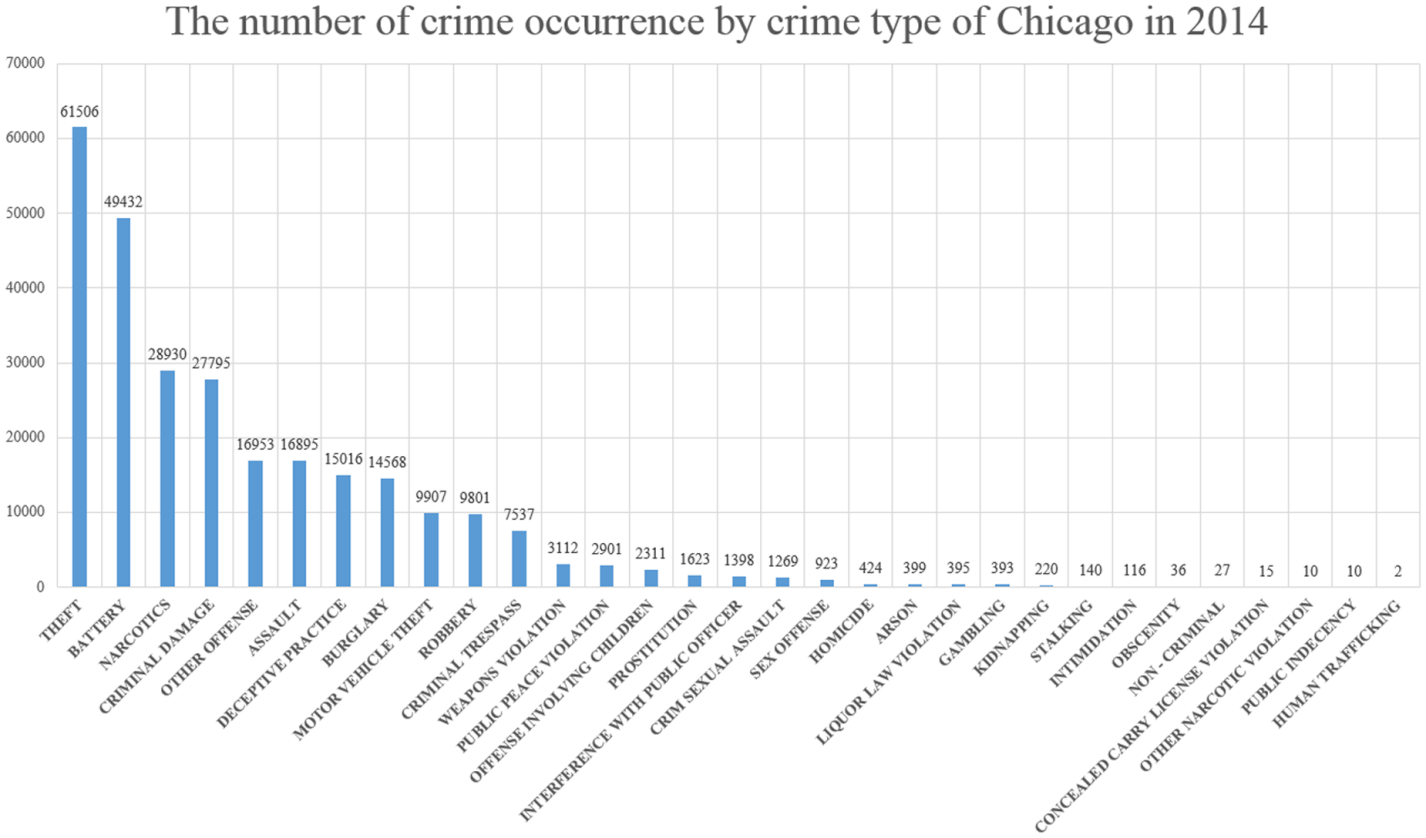
Number of incidents of crime occurrence by crime type of Chicago in 2014. The crime occurrence report data of Chicago in 2014 has a total of 274,064 cases of 31 crime types.

Data collection is critical for the accurate prediction of crime occurrences. In this section, we present collection methods for data from Chicago, Illinois. We employed data from seven domains: crime occurrence reports, demographic, housing, economic, education, weather, and image data. Data were collected from Chicago because it has both a large population (approximately 2.7 million) and a high crime level (a total of 274,064 cases in 2014). The report containing crime occurrence data was collected from the City of Chicago Data Portal. We used the report from 2014, which contains the date, crime type, and latitude/longitude coordinates of incidents involving crime. The report lists a total of 274,064 cases of 31 crime types. Fig 3 shows the number of incidents of crime occurrence by crime type for Chicago in 2014.

In addition, we used 2014 American Community Survey (ACS) data from American FactFinder (http://factfinder.census.gov) to collect various socioeconomic factors (demographic, housing, education, and economic data for Americans), which are organized in the census tract scale of Chicago. Finally, we eliminated 11 of 801 census tracts that had incomplete data. For example, census tracts 9800 and 9801 have no data and census tracts 3406, 3501, 3504, 3805, 3815, and 3817 lack median housing value data.

**Fig 4:**
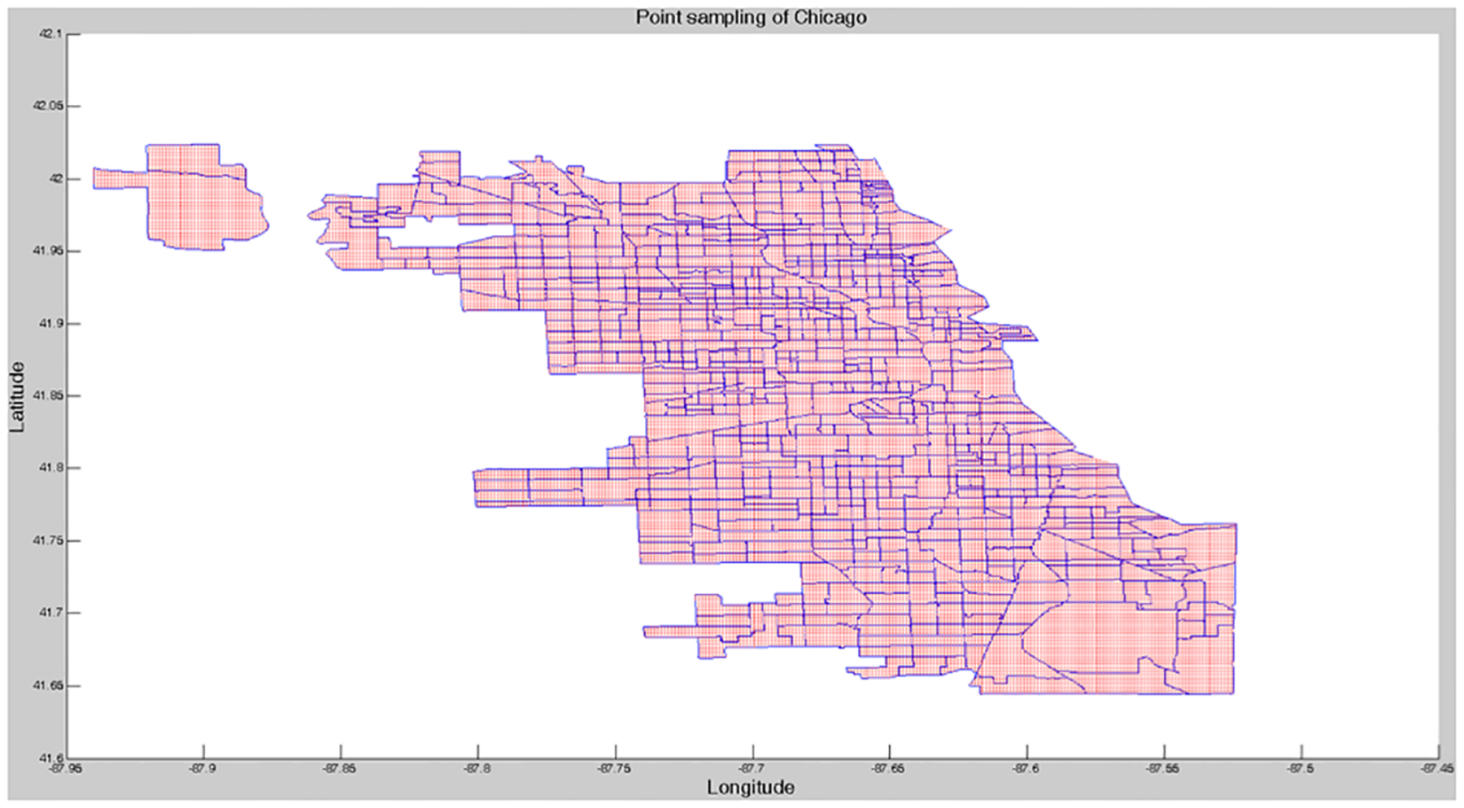
Result of point sampling of Chicago. We acquired every 0.001 latitude/longitude coordinates within the boundaries of Chicago. Blue lines and red dots denote the census tract of Chicago and sampling points, respectively.

We collected weather and image data by using the Weather Underground API (https://www.wunderground.com/) and the Google Street View Image API (https://developers.google.com/maps/documentation/streetview/), respectively. Weather data were captured from the daily weather history of Chicago. These consisted of the mean, maximum, and minimum values of weather and weather events (e.g., snow, rain, hail, and tornados). We eliminated mean humidity and snowfall data with missing values. Moreover, we eliminated hail and tornados, which did not occur in 2014. Image data were collected using latitude/longitude coordinates. They were obtained by using point sampling within the boundaries of Chicago. The data were acquired for all 0.001 latitude/longitude coordinate increments within the boundaries of Chicago: [41.644, -87.940] to [42.023, -87.524], excluding the eliminated census tracts (*n* = 60,348). Fig 4 shows the results of point sampling for Chicago. However, a problem arose: for some sampling points, an image did not exist because the coordinates of the sampling point were discrete. We overcame this problem by using the Google Maps Geocoding API (https://developers.google.com/maps/documentation/geocoding/intro) for sampling points for which an image did not exist. We first converted the coordinates of the sampling point to the corresponding address, and then converted the address to the new corresponding coordinates. In this way, the converted coordinates were provided with image data. After this process, some coordinates still did not have corresponding images. Nevertheless, it was possible to reduce the number of coordinates for which an image did not exist (before processing: 15,624; after processing: 4,910). Finally, we eliminated the missing values from each dataset. The dataset we collected is S1 and S2 Files. Table 1 presents a summary of our datasets.

**Table 1:** Summary of our datasets.

Domain	Collection	Collected data	Scale
Demographic	American FactFinder	- 10 data related to the population. - 8 data related to the race.	Census tract
Housing	American FactFinder	- 6 data related to housing.	Census tract
Education	American FactFinder	- 12 data related to education.	Census tract
Economic	American FactFinder	- 14 data related to employment. - 26 data related to industry.	Census tract
Image	Google Street View	- 60,348 images (640 x 420).	Coordinates
Weather	Weather Underground	- 20 data related to the weather. - 6 data related to weather events	Date

Census tract 705, 711, 3406, 3501, 3504, 3805, 3815, 3817, 8357, 9800, and 9801 did not collect because they does not have some data. Mean humidity and snowfall are missing value. Hail and tornado did not occur.

## Data selection

**Fig 5:**
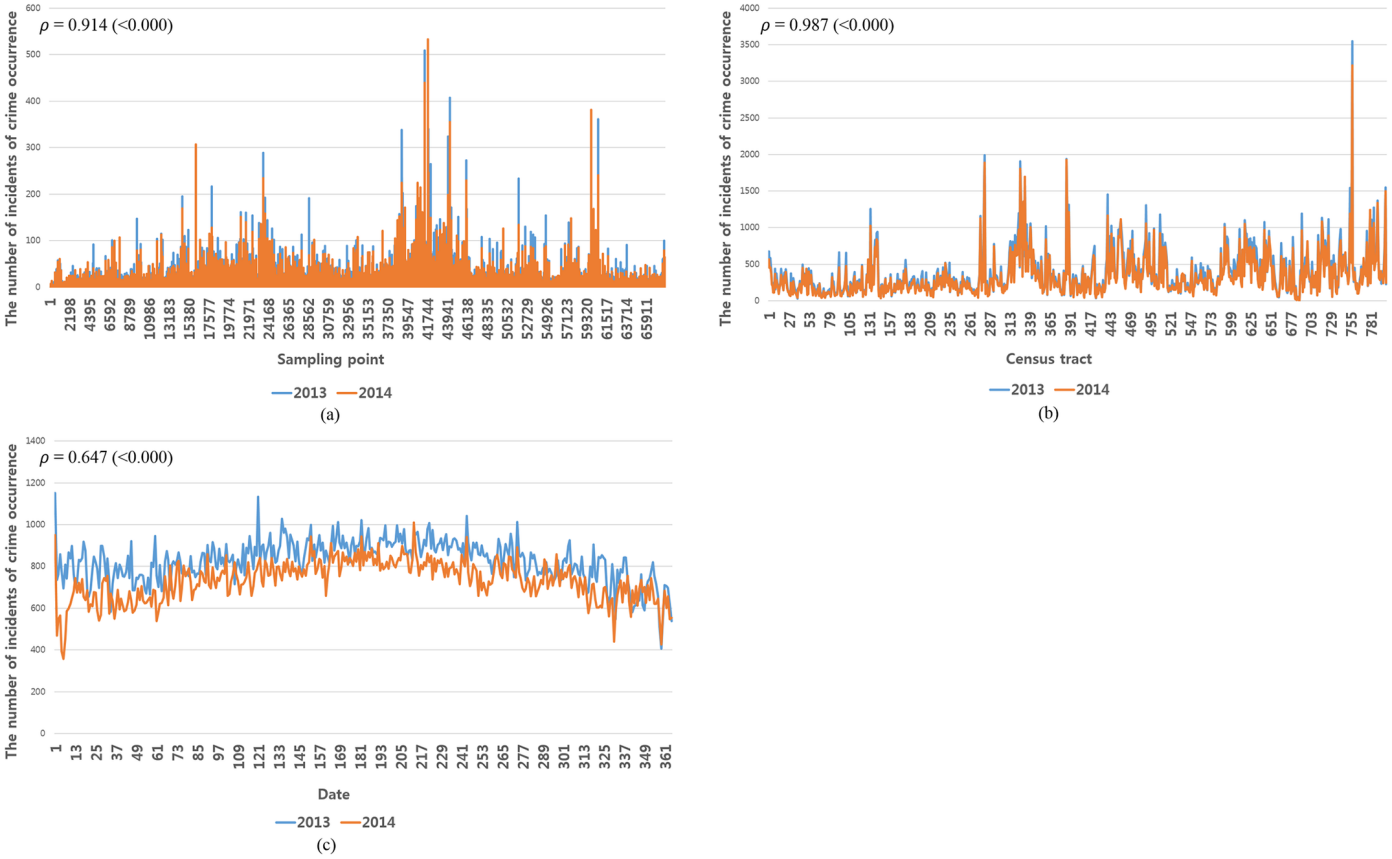
Comparison of the number of incidents of crime occurrence in Chicago by sampling point, census tract, and data in 2013 and 2014. (a) by sampling point, (b) by census tract, and (c) by date. (*ρ* denotes the Pearson correlation coefficient).

The collected data from various online databases may contain information which does not need for crime occurrence. To solve this, it is necessary to eliminate anomalies and outlying data for selecting meaningful data with statistical significance related to crime occurrences. This filtering facilitated accurate and effective prediction of crime occurrences. To this end, we conducted Pearson correlation coefficient analysis. However, the crime occurrence report uses coordinates for its scale; nevertheless, the demographic, housing, education, and economic data use the census tract as their scale. In addition, weather data are scaled according to the date. That is, each data resource has a different data scale for crime occurrences, which required us to conduct alignment processing on the various data scales. From that point, we conducted Pearson correlation coefficient analysis. In addition, to analyze temporal crime occurrence patterns, such as the initial crime location, with a high probability of occurrence of the same type of crime [6] and the relationship between the number of crime incidents in 2013 and 2014, we conducted another Pearson correlation coefficient analysis. Fig 5(a), 5(b) and 5(c) compare the number of incidents of crime in Chicago by sampling points, census tracts, and dates in 2013 and 2014. We used the statistical analysis software package SPSS 18.0 to conduct the Pearson correlation coefficient analysis. Finally, we regarded data with a Pearson correlation coefficient in the range from -0.2 to 0.2 and with a p-value greater than 0.05 as noisy data and discarded them. The Pearson correlation coefficient results in S3 File indicate that 53 out of 102 items of information have a correlation with a crime occurrence.

However, the environmental context information could not be captured from the analysis of the Pearson correlation coefficient because it is a 4096-D feature vector extracted from Alexnet using image data. Thus, to analyze the difference in the number of crime incidents according to environmental context information, we conducted a Kruskal-Wallis H test (also known as a “one-way ANOVA on ranks”), which is a rank-based non-parametric test to analyze statistically significant differences between two or more independent groups. Environmental context information had to be grouped to conduct the Kruskal-Wallis H test because it did not assume a normal distribution. Each group should have been composed of environmental context information that is similar to appearance. We achieved this objective by dividing the environmental context information into ten groups and using k-means clustering to conduct the Kruskal-Wallis H test and Dunn’s test with Bonferroni-type adjustment of p-values for a post hoc test after the Kruskal-Wallis H test. The pairwise multiple comparisons of mean rank sums (PMCMR) package in the R software package were employed to conduct the above noted tests. Tables 2 and 3 present the results of the Kruskal-Wallis H test and Dunn’s test with Bonferroni-type adjustment of p-values for the post hoc test after the Kruskal-Wallis H test. The results of the Kruskal-Wallis H test show a p-value of less than 0.05. In addition, the results of Dunn’s test with Bonferroni-type adjustment of p-values for the post hoc test after the Kruskal-Wallis H test show differences in statistical significance between the environmental context information groups. These results demonstrate that a difference exists in the number of crime incidents according to environmental context information. In other words, it is appropriate to use environmental context information to predict the occurrence of a crime.

**Table 2:** Results of Kruskal-Wallis H test.

**Chi-square**	542.667
**Degree of freedom**	9
**P-value**	<0.001

**Table 3:** Results of Dunn’s test with Bonferroni-type adjustment of p-values for post hoc test after Kruskal-Wallis H test.

**p-value**	**Cluster1**	**Cluster2**	**Cluster3**	**Cluster4**	**Cluster5**	**Cluster6**	**Cluster7**	**Cluster8**	**Cluster9**
**Cluster2**	<0.001	-	-	-	-	-	-	-	-
**Cluster3**	<0.001	<0.001	-	-	-	-	-	-	-
**Cluster4**	<0.001	1.000	<0.001	-	-	-	-	-	-
**Cluster5**	<0.001	<0.001	1.000	<0.001	-	-	-	-	-
**Cluster6**	<0.001	<0.001	0.001	0.006	<0.001	-	-	-	-
**Cluster7**	<0.001	1.000	<0.001	1.000	<0.001	0.104	-	-	-
**Cluster8**	<0.001	<0.001	0.069	<0.001	<0.001	1.000	<0.001	-	-
**Cluster9**	<0.001	<0.001	1.000	<0.001	1.000	<0.001	<0.001	<0.001	-
**Cluster10**	<0.001	<0.001	0.034	<0.001	<0.001	1.000	0.003	1.000	<0.001

## Prediction model

In this section, we describe the structure and learning method of our prediction model. We employed DNN using feature-level data fusion from three disparate feature groups: spatial, temporal, and environmental contexts. The three feature groups were clustered from collected data.

### DNN-based prediction model with feature-level data fusion

Recently, a DNN has been used to learn joint feature representations from multiple datasets in a multi-modal data fusion approach. That is, a DNN learns how to integrate features into a unified feature. Thus, a DNN-based prediction model with feature-level data fusion method generally performs more satisfactorily than a direct concatenation method because it can overcome the limitations of the latter method; i.e., over-fitting, difficulty discovering highly nonlinear relationships, and redundancy and dependency between multiple datasets [21, 35, 37]. Therefore, we employed a DNN-based prediction model with a feature-level data fusion method for the prediction of crime occurrences.

**Fig 6:**
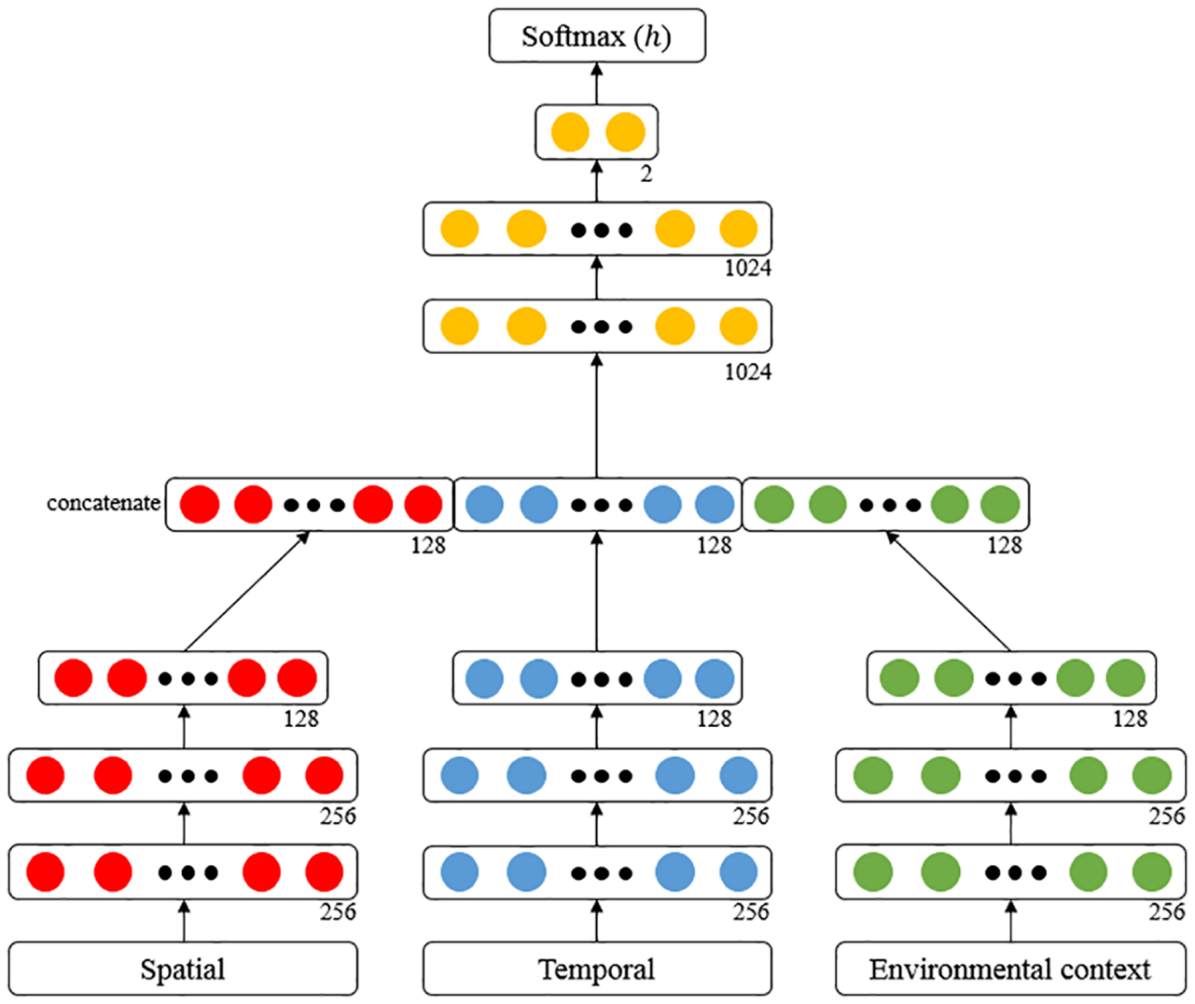
The structure of our DNN. It consists of spatial feature, temporal feature, environmental context feature, joint feature representations layers and softmax classifier.

Fig 6 shows the structure of our DNN. We configured the DNN with four layers: spatial, temporal, environmental context, and joint feature representation layers. First, spatial, temporal, and environmental context feature layers operate independently. Each feature layer uses the corresponding feature group consisting of data with properties similar to its input to perform multi-level feature representation and abstraction. The feature layers play a role in extracting features from the input information. The results of these three feature layers were concatenated and then provided into the joint feature representation layer to integrate the features into a unified feature. The joint feature representation layer learns the appropriate weights in order to integrate the three features. The spatial, temporal, and environmental context feature layers consisted of three layers with 256, 256, and 128 neurons, respectively. The joint feature representation layer had three layers with sizes of 1024, 1024, and 2. All layers applied rectified linear units for activation functions and dropouts. Because our task involved binary classification, we used SoftmaxWithLossLayer in a Caffe framework [38] as the loss layer. This computed the multinomial logistic loss for a one-of-many classification task, passing real-valued predictions through Softmax to obtain a probability distribution over classes. The loss *E* is computed using the following equations:

$$\begin{array}{c} p_{\left(n,k\right)}=\frac{\exp(x_{n,k})}{\sum_{k^{\prime }=0}^{K-1}\exp(x_{n,k^{\prime }})}, \end{array}$$ (1)

$$\begin{array}{c} E=\frac{-1}{N}\sum_{n=1}^{N}\log\left(p_{n,l_{n}}\right), \end{array}$$ (2)

where *N* and *K* denote the batch size and class, respectively, and *p*_(*n*,*k*)_ denotes the Softmax output class probability.

### Learning

We generated a training dataset by conducting point sampling for all 0.001 latitude/longitude coordinate increments for Chicago, excluding census tracts 705, 711, 3406, 3501, 3504, 3805, 3815, 3817, 8357, 9800, and 9801. The sampling points were acquired every day from January 1, 2014 to December 31, 2014 (*m* = 60,348 * 365). We then mapped the location of daily crime occurrences to the nearest points. We labeled the sampling points as either crimes or non-crimes according to whether daily crimes occurred.

**Fig 7:**
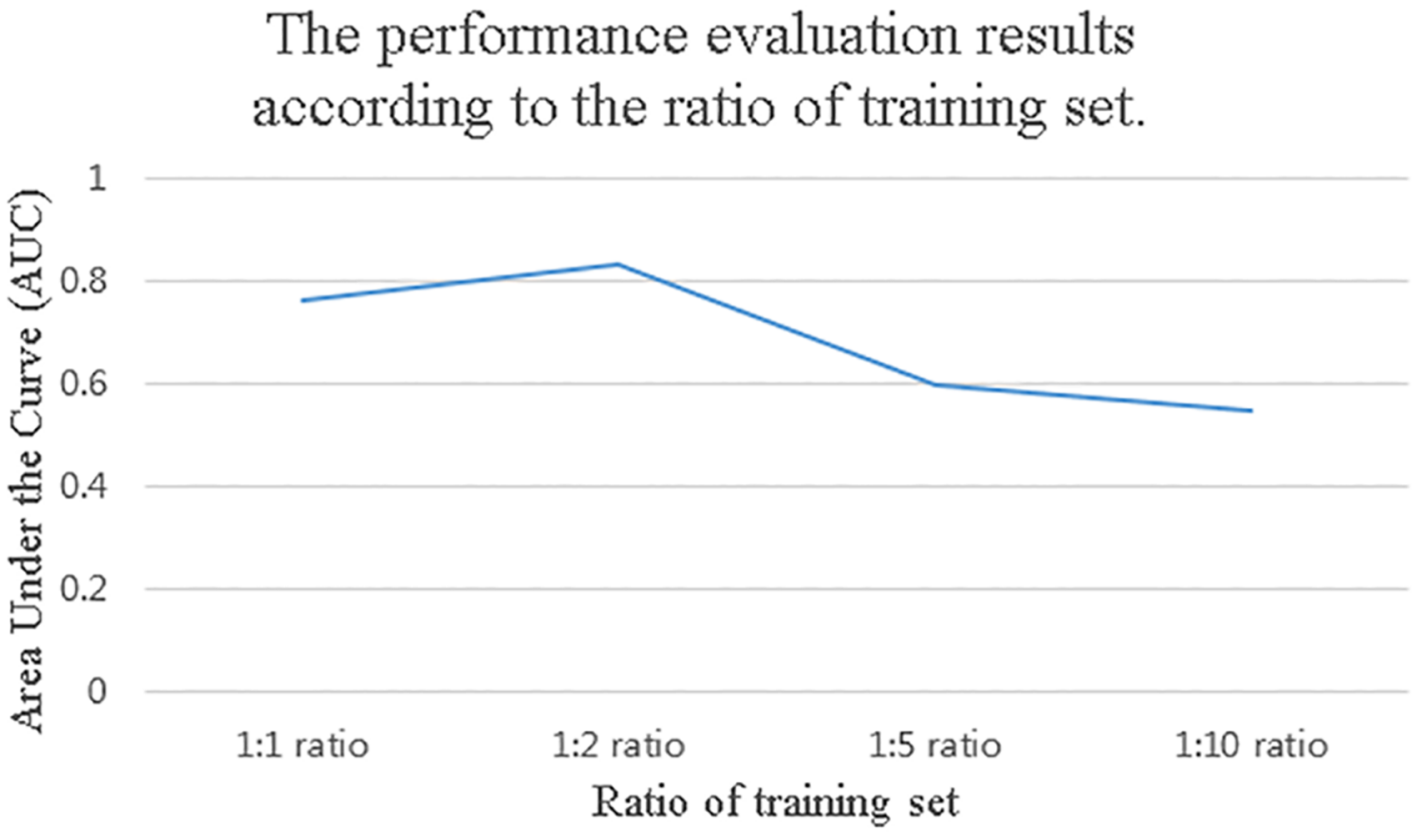
Performance evaluation results according to the ratio of training set. We performed evaluation for 1:1, 1:2, 1:5, and 1:10 ratio.

However, this method reached an imbalance between crimes and non-crimes on account of the overwhelming lack of crime occurrence report data at all sampling points (the ratio of crime points to non-crime points was 264,117:21,827,143; i.e., the crime rate was approximately 1%). This imbalanced data can cause significant performance reduction for the prediction model [39, 40]. We solved this problem by randomly under-sampling the training non-crime points from all the daily non-crime points. We used the training non-crime points as twice the number of daily crime points (this ratio was obtained empirically, as indicated in Fig 7). For example, if the number of crime points was 300 on January 1, 2014, then we randomly extracted 600 non-crime points from all the non-crime points on January 1, 2014.

Next, we classified the data into the three independent feature groups mentioned above, namely spatial, temporal, and environmental contexts, according to the data properties. The data groups were as follows:

- Spatial feature group (35-D): demographic (9-D), housing (6-D), education (8-D), and economic (12-D).
- Temporal feature group (15-D): weather (11-D), number of incidents of crime occurrence by sampling point in 2013 (1-D), number of incidents of crime occurrence by census tract in 2013 (1-D), number of incidents of crime occurrence by date in 2013 (1-D), and number of incidents of crime occurrence by census tract yesterday (1-D).
- Environmental context feature group (4096-D): an image feature (4096-D).

We trained the DNN by using the deep learning framework Caffe [38], which provides simple and powerful parallel computing for DNN learning. We set the batch size, initial learning rate, and dropout rate to 256, 0.01, and 0.5, respectively.

## Experimental results and discussion

### Prediction performance

We analyzed the performance of our prediction model by comparing it with SVM and KDE. SVM is a general machine learning framework. We trained the SVM using LIBLINEAR [41], which is a publicly available library for large linear classification. The SVM was trained using a unified feature set, which was generated by direct concatenation. We trained while varying the parameter *c* to obtain the optimal value. A KDE-based prediction model, which is a general method to estimate crime occurrence densities, was trained by using the *ks* package in the R software package, and we obtained the optimal value of the parameter *h* using the plug-in bandwidth with the *dscalar* pilot. We then measured the performance of the prediction model by calculating the accuracy, precision, recall, and area under the curve (AUC). We evaluated performance according to the training data used with the DNN and the three independent classifiers of our DNN model. Table 4 presents the performance evaluation results of our prediction model and the other two models. From these results, we found that our DNN-based multi-modal data fusion method is a more appropriate method of predicting crime occurrence than the previously proposed direct concatenation or the probabilistic approach method, both of which demonstrated low efficiency.

**Table 4:** Performance evaluation results according to the ratio of training set.

Model	Accuracy (%)	Precision (%)	Recall (%)	AUC
**SVM**	67.01	98.58	1.05	0.5052
**KDE**	66.33	43.04	85.79	0.7258
**DNN (our method)**	84.25	74.35	80.55	0.8333

### Training set ratio

Our prediction model was trained on a 1:2 (crime:non-crime) training set because of imbalanced data. We employed a random under-sampling approach but do not know the appropriate ratio. Therefore, we empirically obtained the appropriate ratio of crime:non-crime. We evaluated the performance of the prediction model while changing the crime:non-crime ratio of the training set as 1:1, 1:2, 1:5 and 1:10 in Fig 7. We evaluated our method across 5 random train/test splits for each ratio of training set. Although the 1:2 ratio required slightly more learning time than the 1:1 ratio (1:1 at approximately 2.5 hours and our DNN at approximately 4 hours), our DNN was more accurate than the DNN with a 1:1 ratio. Therefore, we determined that the generation of training data is important for the accurate prediction of crime occurrences.

In fact, we trained the prediction model using all sampling points (with a crime:non-crime ratio of 264,117:21,827,143), which is not shown in Fig 7. The results of the learning prediction model using all sampling points show that incorporating all the data is indeed inefficient. While the learning time is increased by approximately 20 times, the prediction performance remains very low on account of the imbalance between the numbers of crimes and non-crimes in the training set.

### Effect of data selection

In general, high-dimensional data can present prediction difficulties and increase computational costs, such as by the ‘curse of dimensionality.’ Moreover, using all the collected data may lead to degrade performance due to redundancy of data, noisy data, or non-related data. We overcame this problem by utilizing effective statistical analysis to encompass the important data for identifying major trends. As shown in Table 5, using a DNN with data selection (4149-D) improves performance compared to using a DNN without data selection (4198-D). However, we did not obtain a great reduction in the number of dimensions because of the high dimensionality of environmental context information. Nevertheless, we considered this to be a useful approach in cases with immense datasets (i.e., we did not obtain a reduction in the computational cost).

**Table 5:** Results of performance evaluation according to the used data.

Model	Accuracy (%)	Precision (%)	Recall (%)	AUC
**DNN (our method)**	84.25	74.35	80.55	0.8333
**DNN without data selection**	77.98	70.69	57.96	0.7297
**DNN without environmental context information**	72.38	59.96	51.58	0.6718

### Environmental context information

We demonstrated the relationship between environmental context information and crime occurrence through k-means clustering, a Kruskal-Wallis H test, and Dunn’s test with Bonferroni-type adjustment of p-values for a post hoc test after the Kruskal-Wallis H test. The statistical test results showed that a difference exists in the number of crime incidents according to the environmental context information group. In practice, we showed a performance difference according to the environmental context information in Table 5. Hence, we analyzed environmental context information by comparing the groups with the highest and lowest mean crime occurrences. We found that the group with the highest mean appears in areas with modern buildings in urban environments; i.e., districts with a large floating population. On the other hand, the group with the lowest mean appears in areas consisting of fields and mountains; i.e., districts with a small floating population. This result underscores the difference in the number of crime incidents according to environmental context information. We also analyzed the effect of environmental context information for the prediction of crime occurrence by training the DNN model with and without environmental context information. The evaluation results presented in Table 5 confirm that we demonstrated the importance and usefulness of environmental context information (image data) for the accurate prediction of crime occurrences.

### Visualization

**Fig 8:**
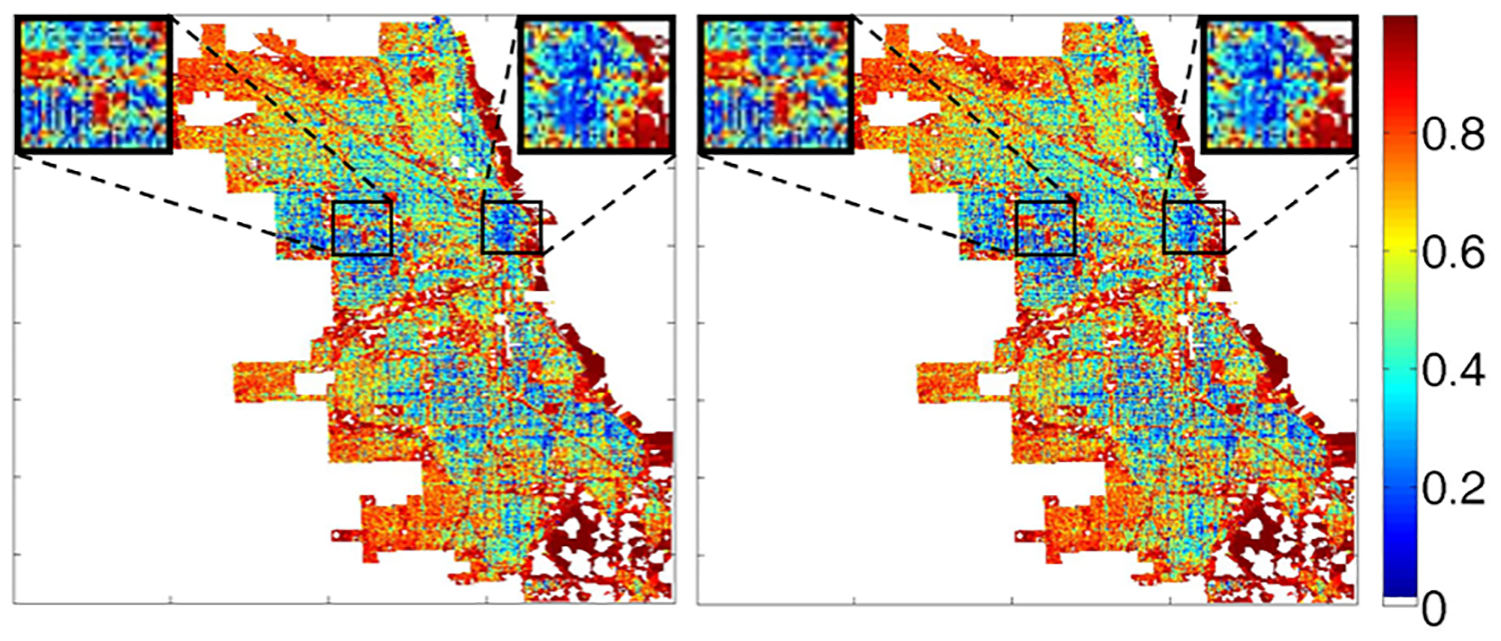
Example of visualization results for the predicted crime occurrence probability at Dec. 25 (left) and Dec. 26 (right) in 2014. The probability is depicted in numerical form as a heat map. Black lines denote the boundary of census tract of Chicago. Census tracts 705, 711, 3406, 3501, 3504, 3805, 3815, 3817, 8357, 9800, and 9801 are blank because they does not have some data.

The output of our crime occurrence prediction model is the probability of crime and non-crime occurrences using sampling points. Because these outputs are numerical, it is difficult to intuitively understand and utilize them. To make the predicted probability of crime occurrence more readily understandable, effective visualization is needed. Visualization is a more accessible visual representation of data for seemingly abstract and/or abstruse data. In our case, we encoded the predicted probability of crime occurrences for easier understanding and utilization in the effective implementation of predictive police patrols. To effectively visualize the predicted probability of crime occurrences, we represented the predicted probability of crime occurrences as a heat map. Fig 8 shows an example of a visualization result for the predicted crime occurrence probability for Dec. 25 and Dec. 26 in 2014. Even though the actual crime occurrence patterns do not change much, we can detect slight differences between two images. Although we did not include a method of generating a recommended police patrol path, the visualization of crime probabilities can ameliorate the decision making involved in predictive police patrols. Moreover, hotspot patrols can be made more effective by using the represented probabilities of criminal activities occurring in specific areas at certain times.

### Limitation

The limitation of our study is that we cannot apply our DNN-based crime occurrence prediction method to regions with insufficient data. This lack of data in certain areas may lead to significant performance degradation. In particular, in the absence of crime occurrence report data, crime occurrence prediction is nearly impossible. Furthermore, our present crime occurrence prediction method is unable to provide information regarding a specific crime type at a given time slot.

## Conclusion

In this paper, we proposed an accurate crime occurrence prediction method by efficiently fusing multi-modal data from multiple domains with environmental context information. Our method incorporates past criminal activity records in certain areas and models them based on deep learning to predict the occurrence of crimes. Police patrols can leverage predictive crime information to more effectively monitor crime hotspot areas and improve the overall effectiveness of police patrols. Our approach consisted of three phases. First, we collected various types of data from the City of Chicago Data Portal, American FactFinder, Weather Underground, and Google Street View. Image data were used to extract environmental context information. We then analyzed the relationship between crime incidents and the collected data using a statistical approach. We thereby generated a dataset that can be used more effectively in crime prediction. Finally, to accurately predict crime occurrences, we employed a DNN using feature-level fusion with different weights to efficiently proportion the data in order to integrate spatial, temporal, and environmental context features.

As a result, our DNN, which used a 1:2 ratio of training data generation obtained empirically, shows an accuracy of 84.25, precision of 74.35, recall of 80.55, and AUC of 0.8333. All of these values are higher than the corresponding values produced by the traditional methods. In addition, we compared our DNN with some models that used different training datasets and demonstrated the effect of our data selection and environment context information process. Our DNN-based fusion model, coupled with environmental context information, is thus productive in crime prediction. Furthermore, we visualized the predicted crime occurrence probability as a heat map to help elucidate the seemingly compact and abstract results.

The limitation of our study is that our DNN-based method for the prediction of crime occurrences cannot be applied when sufficient data is unavailable. Therefore, we aim to solve this problem using a machine-learning algorithm, such as transfer learning or co-training. We additionally aim to evaluate the performance of a real-world application using our prediction model.

Furthermore, we plan to extend this study to predict the type and time slot of crime occurrences and to find other data for their prediction. Moreover, we intend to develop a method of generating an effectively predictive police patrolling path from the predicted crime occurrence probability to enhance the efficiency of the police patrolling system.

## Supporting information

S1 File Crime occurrence reports, demographic, housing, economic, and education datasets. (XLSX)

S2 File Weather dataset. (XLSX)

S3 File Results of Pearson correlation coefficient analysis. (XLSX)
